# Uremia with War Wounded

**Published:** 1919

**Authors:** 


**Uremia with War Wounded.** By H. Reynes. *La Presse Médicale*, October 10, 1918.

Ever since January, 1916, M. Henry Reynes has maintained that serious war wounds constitute dangerous seats of cellular attrition. In these seats, where local life is compromised or even suppressed, the organic elements undergo a process of regressive disintegration, become toxic and are carried in the whole system, which they poison. Among these toxic elements urea is the most easily detected. With all seriously wounded there is an immediate and marked increase of urea within 24 hours. If the percentage is high, uremia takes place, owing to incomplete evacuation. During the "ureic tension" the best treatment is to give the patients hot sweetened drinks, injections of glycosed serum, theobromine and lactose. Stewed fruit may be given, but no nitrogenized food, and no milk whatever.

The uropoietic function of the digestive tube should be enticed by repeated absorption of purgative salts. It is well also to make the patient warm by rubbing, friction, and wrapping in cotton wool. In the case of uremia, bleeding is indicated, rhachidian punctures, and intravenous injections of glycosed serum.

For local treatment, trim the dead tissues, and practise disinfection by large and free washing.

The uro-pronostic is scientifically and practically very important. Many a time it has saved the life of wounded more endangered by intoxication than by the actual evolution of the wound.

